# Single amino acid (arginine) deprivation: rapid and selective death of cultured transformed and malignant cells

**DOI:** 10.1054/bjoc.2000.1353

**Published:** 2000-08-17

**Authors:** L Scott, J Lamb, S Smith, D N Wheatley

**Affiliations:** Department of Cell Pathology, University of Aberdeen, MacRobert Building, 581 King Street, Aberdeen, AB24 5UA, UK

**Keywords:** arginine, growth, death, malignant cells, normal cells

## Abstract

The effects of arginine deprivation (–Arg) has been examined in 26 cell lines. Less than 10% of those with transformed or malignant phenotype survived for > 5 days, and many died more rapidly, notably leukaemic cells. Bivariate flow cytometry confirmed that vulnerable cell lines failed to move out of cell cycle into a quiescent state (G0), but reinitiated DNA synthesis. Many cells remained in S-phase, and/or had difficulty progressing through to G2 and M. Two tumour lines proved relatively ‘resistant’, A549 and MCF7. Although considerable cell loss occurred initially, both lines showed a ‘cell cycle freeze’, in which cells survived for > 10 days. These cells recovered their proliferative activity in +Arg medium, but behaved in the same manner to a second –Arg episode as they did to the first episode. In contrast, normal cells entered G0 and survived in –Arg medium for several weeks, with the majority of cells recovering with predictable kinetics in +Arg medium. In general, cells from a wide range of tumours and established lines die quickly in vitro following –Arg treatment, because of defective cell cycle checkpoint stringency, the efficacy of the treatment being most clearly demonstrated in co-cultures in which only the normal cells survived. The findings demonstrate a potentially simple, effective and non-genotoxic strategy for the treatment of a wide range of cancers. © 2000 Cancer Research Campaign


*British Journal of Cancer* (2000) **83**(6), 800–810
© 2000 Cancer Research Campaign
doi: 10.1054/ bjoc.2000.1353, available online at http://www.idealibrary.com on IDEAL®

### Single amino acid (arginine) deprivation: rapid and selective death of cultured transformed and malignant cells

**L Scott, J Lamb, S Smith and DN Wheatley**

Department of Cell Pathology, University of Aberdeen, MacRobert Building, 581 King Street, Aberdeen AB24 5UA, UK

**Summary** The effects of arginine deprivation (–Arg) has been examined in 26 cell lines. Less than 10% of those with transformed or malignant phenotype survived for > 5 days, and many died more rapidly, notably leukaemic cells. Bivariate flow cytometry confirmed that vulnerable cell lines failed to move out of cell cycle into a quiescent state (G0), but reinitiated DNA synthesis. Many cells remained in S-phase, and/or had difficulty progressing through to G2 and M. Two tumour lines proved relatively 'resistant', A549 and MCF7. Although considerable cell loss occurred initially, both lines showed a 'cell cycle freeze', in which cells survived for > 10 days. These cells recovered their proliferative activity in +Arg medium, but behaved in the same manner to a second –Arg episode as they did to the first episode. In contrast, normal cells entered G0 and survived in –Arg medium for several weeks, with the majority of cells recovering with predictable kinetics in +Arg medium. In general, cells from a wide range of tumours and established lines die quickly in vitro following –Arg treatment, because of defective cell cycle checkpoint stringency, the efficacy of the treatment being most clearly demonstrated in co-cultures in which only the normal cells survived. The findings demonstrate a potentially simple, effective and non-genotoxic strategy for the treatment of a wide range of cancers. © 2000 Cancer Research Campaign

**Keywords:** arginine; growth; death; malignant cells; normal cells

Having investigated all the essential amino acids, withdrawal of arginine (–Arg) was least tolerated by tumour cells (HeLa) in vitro (Wheatley et al, 2000). Because Arg cannot normally be synthesized by cultured cells, it is an essential amino acid. Most essential amino acids have relatively uncomplicated metabolisms related principally to protein synthesis, as typified by Leu (Neff et al, 1977). However, the rapid metabolism of Arg through many different pathways reduces its availability in culture faster than other amino acids (Wheatley et al, 2000; see also Discussion). Furthermore, Arg is less efficiently re-utilized from catabolized proteins than Leu or Lys (Wheatley et al, 2000). Its withdrawal also leads to a more flagrant 'pleiotypic response' (Rabinovitz, 1992) than found with deficiencies of other amino acids. This greater 'demand' on Arg is still not fully appreciated by cell culturists (see Daniel and Krishnan, 1968; Paul and Walters, 1974), although Eagle (1955; 1959) did note that his chosen cell lines required additional Arg supplementation in the early 'optimal growth' studies. Indeed, the arginine problem is better appreciated in whole animal growth studies (Wu and Morris, 1998).

Removal of one or more of any of the 11 essential amino acids might be expected to produce similar biological end-points in cell cycling both of normal and malignant phenotypes, but this is not so. A rapid demise of tumour cells occurs in Arg-free (–Arg) medium, whereas diploid fibroblasts move into quiescence, a differential which clearly offers a selective advantage in targeting tumour cells (Wheatley et al, 2000). Tumour cells, unlike their normal counterparts, initiate further rounds of replication following deprivation and fail to enter quiescence, as seen with KB (a human cervical carcinoma cell line; Weissfeld and Rouse, 1977*a–c*). For this reason they cannot be synchronized by this procedure, whereas normal cells and occasionally others can, e.g. CHO, a fibroblastic cell line from the Chinese hamster ovary (Tobey and Ley, 1970).

Synchrony is more often induced in cell culture by reducing serum to low concentration. The culture medium has a fully nutritional complement, but lacks essential growth factors required for cell-cycle progression. Cells arrest at the mid-G1 checkpoint (Pardee, 1974; 1989), where they accumulate and can be released to initiate S-phase synchronously when serum is restored. In contrast, removal of an essential nutrient leaves the growth factors in place, but restricts growth and cell cycling by inhibiting de novo protein synthesis. Malignant and transformed cells lacking stringent G1 control, however, try to continue cycling, a fundamental difference which should be eminently exploitable as a means of selectively killing tumour cells.

In this comparative study of 26 selected cell lines (Table 1), cells of human origin have mostly been used, because the aim will eventually be to test the efficacy of deprivation on primary tumour cell lines taken from patients prior to in vivo treatment. Murine and a few other species have been included because comparative in vivo studies are in progress in animal models, and certain molecular biology experiments are feasible only in special cell lines, e.g. Mv1Lu (mink lung cells; *see* Lamb, 1998). Investigators have already shown the poor tolerance of malignant cells in vivo to Arg deprivation, e.g. in the murine leukaemic cell lines, L5178 (Storr and Burton, 1974) and L1210 (Ormerod et al, 1994). The implications of our present findings with regard to the treatment of human

*Received 18 January 2000*
*Revised 28 April 2000*
*Accepted 11 May 2000*

*Correspondence to*: DN Wheatley

**Table 1** Effect of Arg deprivation of normal and malignant cell lines in culture

| Cell line | Species | Type | Malignant/ normal | Fate in –Arg* | Time of death of culture** |
|---|---|---|---|---|---|
| MG2F | human | adult skin fibroblast | normal | Q (> 20 days) | – |
| FF5/15 | human | neonatal foreskin fibroblasts | normal | Q (> 28 days) | – |
| IBRI3 | human | fibroblasts | normal | Q (> 10 days) | – |
| IBR3G | human | fibroblasts | transformed | 36 h | 6 days |
| HeLa | human | cervical carcinoma | malignant | 36 h | 3 days |
| A431 | human | squamous arcinoma | malignant | 24 h | 3 days |
| MCF7 | human | breast carcinoma | malignant | (?) Q | – |
| ZR75-1 | human | breast carcinoma | malignant | 48 h | 4–5 days |
| PEO | human | ovarian carcinoma | malignant | 48 h | 3–4 days |
| PC3 | human | prostatic carcinoma | malignant | 48 h | 3 days |
| WiDr | human | colon carcinoma | malignant | 36 h | 4–5 days |
| A549 | human | lung carcinoma | malignant | (?) Q | – |
| KHOS/NP | human | osteosarcoma | malignant | 36 h | 5 days |
| U2OS | human | osteosarcoma | malignant | 48 h | 3 days |
| Saos 2 | human | osteosarcoma | malignant | 3 days | 5 days |
| GO-C-CCM | human | glioma/astrocytoma | malignant | 48 h | 4–5 days |
| U-870-MG | human | glioblastoma | malignant | 48 h | 4–5 days |
| HL60 | human | premyelocytic leukaemia | malignant | 24 h | 2 days |
| MOLT4 | human | lymphoblastic leukaemia | malignant | 24 h | 2 days |
| 3T3 (NIH) | murine | fibroblasts | 'normal' (see text) | 3 days | 4–5 days |
| 3T3 (BIBR) | murine | fibroblasts | 'normal' (see text) | 3 days | 4–5 days |
| 3T3 | murine | fibroblasts | 'normal' (see text) | 3 days | 4–5 days |
| 3T3 SV40 | murine | SV40 transformation of above | transformed | 48 h | 3 days |
| B16-F10 | murine | melanoma | malignant | 48 h | 5 days |
| Mv1Lu | mink | lung | 'normal' | Q | – |
| PtK1 | kangaroo rat | kidney epithelial | 'normal' | Q (> 7 days) | – |

Q = quasi-quiescence; *time at which clear evidence of cell death was found; **time at which cells were irrecoverably lost and/or dead cancer will be discussed, especially since arginase as a means for lowering Arg has been considered for years to be a potential therapeutic method (Bach and Swaine, 1965; Yeatman et al, 1991; Wheatley, 1998). The efficacy of Arg deprivation as an in vivo treatment ultimately depends on the rapidity with which tumour cells in general succumb. We are addressing this problem in vitro initially, in order to identify which tumour types are the more sensitive to deprivation, and also to measure the degree to which resistance types are found. These studies should help to refine an effective Arg deprivation protocol which will lead to a more rational and selective means of tumour therapy in vivo.

### MATERIALS AND METHODS

### Cell lines

HeLa and HeLa S-3, IBR3G and IBR3T, A549, KHOS/NP, MOLT4, HL 60, WiDr, B16-F10, NIH 3T3, GO-G-CCM, U-87-MG, PC3 and A431 cells were purchased from the European Collection of Animal Cell Cultures (Porton Down, Salisbury, UK). Normal human diploid fibroblasts (MG2F and FF) were established from adult skin and neonatal foreskin, respectively, obtained from the surgical theatres at The Royal Aberdeen Hospitals Trust. Mouse fibroblasts (3T3) and SV40 transformed fibroblasts (3T3-SV40) were provided by Professor Anders Zetterberg (Institute of Tumour Biology, Karolinska Institute, Stockholm, Sweden). 3T3/BIBR were obtained originally from the former British Industrial Biological Research Institute (London, UK). Human breast carcinoma cells MCF-7 and ZR 75-1 were generously provided by Professor Arthur Pardee and Dr Debajit Biswas (Dana-Farber Cancer Institute, Boston, MA, USA). The human osteosarcoma cell lines, U20S and Saos-2, were from Dr Lindsey Allen (Imperial Cancer Research Foundation, London, UK), and the human ovarian carcinoma line, PEO1, from Dr Simon Laing (University of Edinburgh, UK). Mink lung epithelial cells (Mv1Lu) were from Dr Mark Ewen (Dana–Farber Cancer Institute), PtK1 cells were provided by Dr Sam Bowser (Wadsworth Laboratories, Empire State Plaza, Albany, NY, USA). All cells tested free of mycoplasm before they were used, and none were cultured beyond five passages from the stock cultures held, which was normally just one passage on from the cells obtained from our sources.

For comparative work, most cell lines were grown in Dulbecco's modified Eagle's medium (DMEM) containing 10% fetal calf serum (FCS) (both GIBCO/BRL), 100 μg $ml^{-1}$ streptomycin sulphate (Sigma) and 100 U $ml^{-1}$ penicillin (Britannia Pharmaceuticals) at 37°C in a humidified atmosphere of 5% $CO_2$. The medium for several cell types, notably the leukaemic types (see Table 1), was RPMI 1649, but growth was not appreciably different in DMEM.

### Arginine deprivation experiments

After exponential growth had been established, cultures were washed with phosphate buffered saline (PBS) and incubated with either complete medium (+Arg) or medium deficient in L-arginine (–Arg), both containing 5% dialysed (> 10 kD) serum (Sigma). Media were prepared in accordance with published formulations, the appropriate amino acids being left out as required.

### Co-cultures

Cultures of mixed tumour and normal cells were set up as indicated in the text. The ratios of the cell types were varied from

© 2000 Cancer Research Campaign 

equivalence up to an excess of tumour cells at a ratio of 8:1, in order to explore the full potential of arginine deprivation on the preferential elimination of tumour cells.

### Cell proliferation

This was monitored by cell counting using a Coulter electronic particle counter. The monolayer was washed with PBS and the cells were detached with 0.2% trypsin (Sigma). The trypsin was neutralized with DMEM containing 10% FCS. In each experiment three samples were counted at each time-point and the mean value ± 1 SD calculated. After periods of deprivation, cell cultures were rescued from –Arg conditions in complete medium containing 10% undialysed FCS to check the recovery kinetics.

### Flow cytometry

Analyses of cell-cycle phase distribution and DNA synthesis (DNA content/BrdU incorporated) were made using the method of Dolbeare et al (1983). Cells were labelled with 100 μM 5-bromo-2′-deoxyuridine (BrdU) (Sigma) for 30 min at 37°C before being fixed in 70% ethanol. Following denaturation with 2 N HCl containing 0.5% Triton X-100 (Sigma), the cells were stained with anti-BrdU-FITC (Becton Dickinson) and propidium iodide (Sigma). DNA content and incorporated BrdU were measured using an EPICS Profile II Coulter electronics flow cytometer, no less than 20 000 events were processed per sample and the bitmap was adjusted to exclude the counting of doublets.

### Cell viability

This was assessed using an MTT assay (Mosmann, 1983). Cells were incubated with 5 mg $ml^{-1}$ 3-[4,5-dimethylthiazol-2-yl]-2,5-diphenyltetrazolium bromide (MTT) (Sigma) for 4 h at 37°C. The cleavage product formazan was then solubilized with 0.04 N HCl in isopropanol for 30–60 min and absorbance was measured on a spectrophotometer with dual filters of 540 nm and 690 nm.

### Electron microscopy

Electron micrographs were obtained conventionally after fixation of cells in medium without serum containing 2.5% glutaraldehyde at room temperature for 20 h, followed by dehydration through to absolute alcohol, Epon embedding and finally sectioning at 800 Å with a Diatome knife. The sections were mounted on uncoated copper grids and stained with lead citrate and uranyl acetate before being viewed at 60 kV in a Jeol 100S electron microscope.

### RESULTS

### Cell proliferation

Growth curves for nine of the cell lines in Table 1 are set out in Figure 1. Although no cells thrive in the absence of arginine, responses showed two distinct patterns. Each of the cell lines shown in Figure 1 has been included, however, because additional features and idiosyncrasies will be discussed below, and/or are the subject of in vivo studies currently in progress. One group of the cell lines arrested and showed little diminution in cell number over the ensuing period of several days to weeks, while the other group showed a rapid loss of cell number, often falling below the initial level and many dropping to near zero within a few days. Normal cell lines fit the former category (Figure 1A; this response is also typical of MG2F, FF, PtK1 and Mv1Lu, which will be discussed below for their own particular experimental value), whereas the overwhelming majority of the malignant or transformed cell types fit the latter category (Figure 1 D–I), with the two notable exceptions (Figure 1B and C). The reason for the ‘resistance’ of MCF7 and A549 cells almost certainly holds the key to an understanding of the underlying molecular disturbances responsible for demise of the sensitive tumour cell lines. The data in Figure 1 does not go beyond 5 days of deprivation, by which time all cells of the susceptible cell lines (Figure 1 D–I) were irrecoverable.

### Cell death

Cell death occurred within the first 5 days of deprivation in the sensitive malignant cell types, many cultures showing complete involution within 3 days, with the leukaemic lines HL60 and MOLT 4 being almost completely obliterated within 2 days (Table 1). Apart from these two, morphological evidence of death was not usually obvious before 24 h, but appeared during the second day of exposure (Figure 2), when cells started rounding up and lifting off the substratum before disintegrating in the culture medium. Their changes were suggestive of lytic cell death rather than apoptosis, and there has been no consistent indication that the latter mechanism is necessarily involved in other cell lines. Closer inspection of the process by electron microscopy reveals that the first changes are in the periphery of the nucleus with more ‘castellation’ of chromatin being observed by about 12–16 h, and with nucleoli becoming more reticular (Figure 2B; *see also* Figure 12 in Wheatley et al, 2000). This is followed at 24 h by small blebs appearing on some cells (Figure 2C), but these blebs resolve and the outer regions of the whole of the cytoplasm take on a more translucent appearance and include more lipid droplets in cells in which the outer membrane has completely smoothed off by 36 h (Figure 2D). Some cells show signs of condensation of the cytomatrix (Figure 2D), but this is not a general feature. Phagosome/lysosome activity is greatly increased from 24–36 h, and by 48 h a mixture of totally dead cells, completely pycnotic cells and some almost normal-looking cells begins to appear (Figure 2E). Thereafter, the cells become increasingly lytic in their cell-death appearance.

Representative examples of the main differences between the normal and malignant cell responses to Arg deprivation are shown in Figure 3. In cultures such as HeLa, ZR 75-1 and KHOS, a few very large (multinucleate) cells were the only survivors after 3–4 days but could not be rescued in +Arg medium by this time.

In ‘normal’ cell lines, quiescence was usually reached within 24–36 h, with only a small increase in cell number during the first day, after which the majority of cells remained intact and apparently viable for several weeks under these conditions, particularly when cultures were given regular changes of –Arg medium. The effects of restoring Arg between days 1 and 7 of deprivation for human diploid fibroblasts and HeLa cells are contrasted in Figure 4. The severity of metabolic depression by cells, measured 3 days after the start of –Arg treatment of several cell lines, was reflected in the MTT test of cellular (mitochondrial) activity (Figure 5), included here because this is a frequently used cytotoxicity assay in tumour cell sensitivity screening. Figure 5 demonstrates its

 © 2000 Cancer Research Campaign

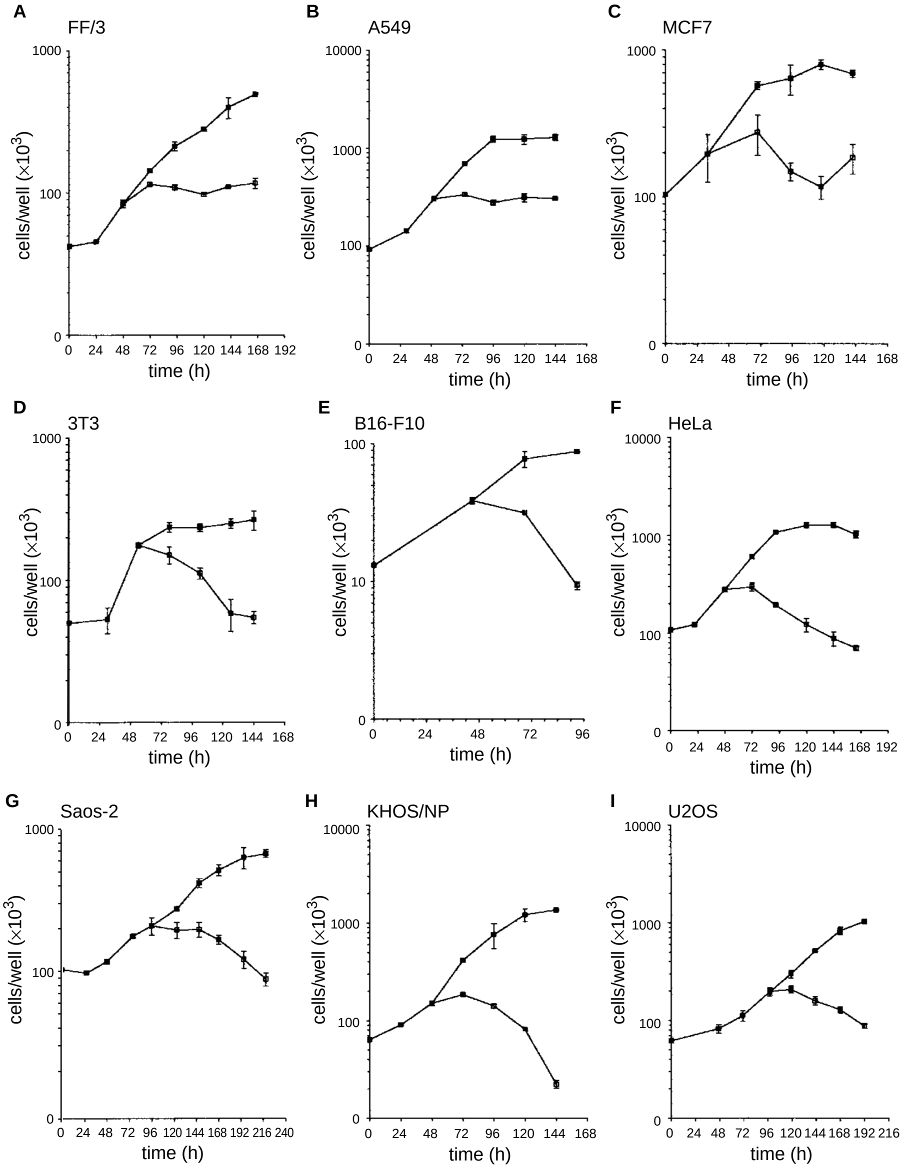

**Figure 1** Growth curves of nine of the 26 cell lines used in this study. They are each representative of the various responses of different cell types to –Arg medium. In the top row, cells in exponential growth were changed to –Arg medium and within ~24 h were downregulated and ceased to proliferate, cell number remaining relatively constant for > 5 days. Much greater variation was found in the cell counting with MCF7 cells (**C**), than in normal fibroblasts (**A**) or A549 cells (**B**). (**B**) and (**C**) show the two 'exceptional examples' of malignant phenotypes responding by downregulation in the face of Arg deprivation (see text), and (**D**)–(**I**) are representative of susceptible mouse and human malignant lines which do show some different kinetics of demise (see text)

value as a quick and effective additional screening method in amino acid deprivation studies on cancer cells; PC3 and H60 cell lines, for example, which rapidly succumb to –Arg, gave MTT OD 540 levels of ~0.05 or less (= 'undetectable') within 3 days. Tumour cells surviving in –Arg medium (A549 and MCF7) show different metabolic activity levels, although most cells left at day 3 were not disintegrating like most other tumour cell lines.

Table 1 shows that the 'matched pair' of established cell lines, IBR3 and IBR3G (the latter being a transformed subline of the former) have quite different fates; the former becomes quiescent, whereas transformed cells die within 5 days (Figure 6). In a similar matched pair comparison, 3T3 cells were found to die relatively quickly in –Arg conditions, being not as fast as its virally transformed subline, 3T3-SV40. NIH 3T3 and BIBR 3T3 were equally sensitive (Table 1, see also Tanaka et al, 1988) to the former cell type. Thus 3T3 cells behave in the manner of transformed lines, unlike IBR3 fibroblasts (see Discussion). The Mv1Lu line was included because transfectants can be made with a truncated cdk4 cDNA which stably express cdk4 (Mv1Lu-B7 and Mv1Lu-H5, Ewen et al, 1995). The parental line can be downregulated in –Arg (Table 1), and behaves like a well-regulated (normal) cell type, whereas the tranfectants do not, showing a cell cycle 'freeze' in –Arg (for data on these transfectants, *see* Lamb, 1998 and Discussion).

### Flow cytometry

Although analyses of 90% of the cell lines used here were performed, Figure 7 gives data on five carefully selected lines which are representative examples of the more interesting changes

*© 2000 Cancer Research Campaign* 

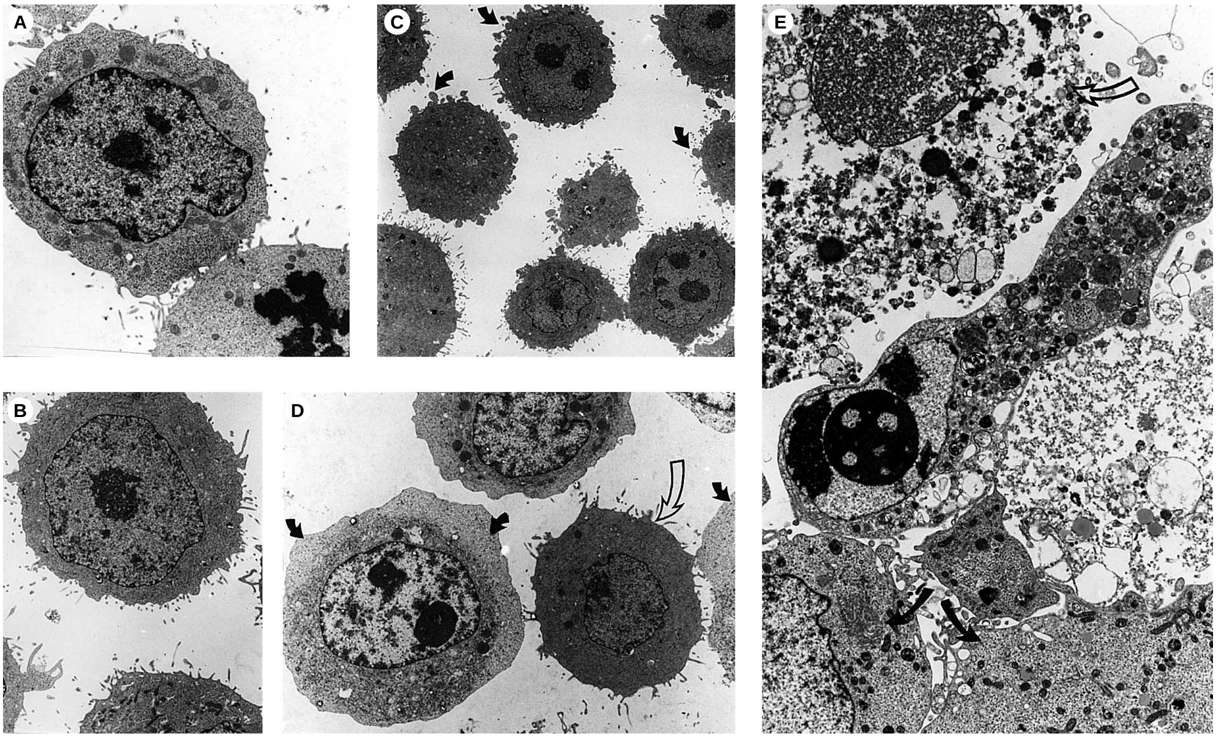

**Figure 2** Transmission electron micrographs of HeLa cells exposed as follows: (**A**) 24 h to +Arg medium (control), (**B**) 24 h to –Arg medium, beginnings of castellation at the nuclear periphery; (**C**) 30 h to –Arg medium, with some surface blebbing (arrows); (**D**) 36 h to –Arg, now with some smoother cell profiles (filled arrows), but with darker smaller cells as well (open arrow); (**E**) 60 h to –Arg; a general view showing the variety of appearances of dead cells, mostly lytic (open arrow), but a few cells retaining more normal appearances (filled arrows). Note the highly pycnotic nucleus in the central cell and the intense lysosomal activity in the cytoplasm. Magnifications: A, B and D: × 4000; C: × 2500; E: × 7500

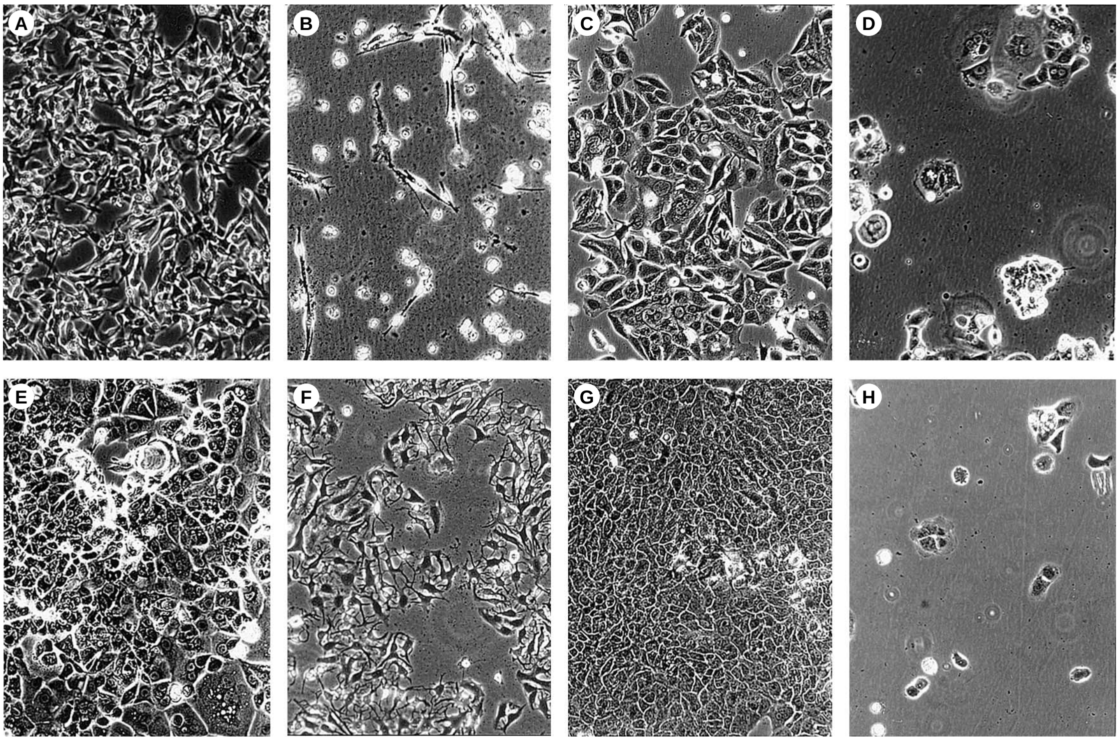

**Figure 3** Photomicrographs of control cultures given +Arg medium (left column) and grown for 3 days compared with parallel cultures given –Arg medium for the same period. (**A**) and (**B**) B16-F10; (**C**) and (**D**) ZR-75-1, note the large multinucleated cells persisting to the right in (**D**), in this cell line 4–5 days are needed before the cells finally die. (**E**) and (**F**) MCF7 cells, with the highly retracted bodies of the deprived cell in the latter frame showing persistence not just for 3 days as here, but for considerably longer than other malignant lines. (**G**) and (**H**) A431 cells, which is a most sensitive cell line. Magnification approximately × 125

 *© 2000 Cancer Research Campaign*

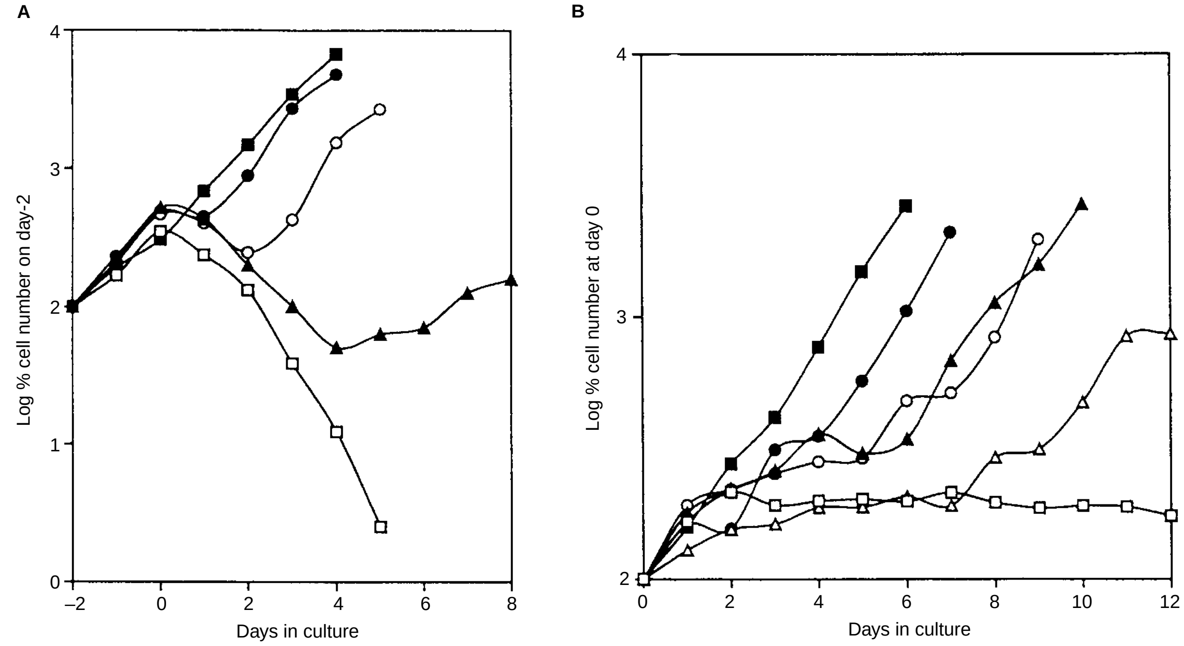

**Figure 4** Graphs showing the degree of recovery of (**A**) HeLa cells or (**B**) normal fibroblasts (MG2F) from exposure to –Arg since the zero-hour time-point until +Arg medium was restored. In (**A**), this was at 0 (■), 1 (●), 2 (○), or 3 (▲) days, with (□) as the non-recovered negative control population. In (**B**) the symbols refer to: 2 (●), 4 (○), and 5 (▲) days of –Arg before recovery was initiated with +Arg medium, and (Δ) shows recovery from 7-day exposure, which was slower than with the shorter exposures.

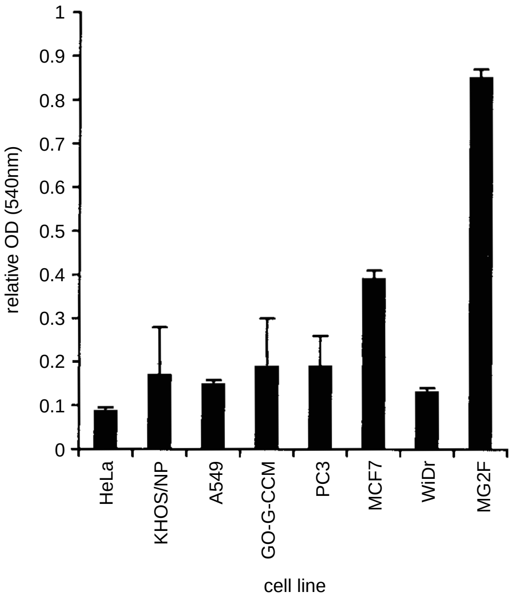

**Figure 5** MTT assays in terms of colour generation per unit mass of cells, with the control (+Arg) value (absorbance at $OD_{540}$ = 0.58) being taken as 100%. The more resistant cells remaining intact (MCF7 and MG2F) show sustained ability to convert the substrate after 3 days in –Arg medium, whereas the malignant cell lines were down to 20% or below the control value. Values are means of six estimates ± 1 SD

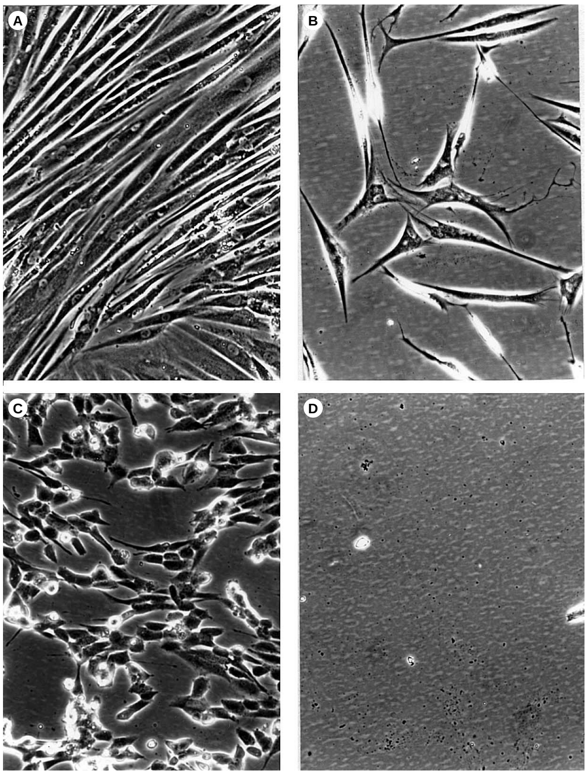

**Figure 6** IBR3 fibroblasts cultured in medium with and without Arg for 3 days. (**A**) Normal IBR3 cells in +Arg; (**B**) Same in –Arg medium; (**C**) the virally transformed subline IBR3G after 3 days in +Arg; (**D**) same after 3 days in –Arg medium. In (**B**) the number of cells present was approximately the same as when the cells were initially set up. Magnification approximately × 200

*© 2000 Cancer Research Campaign* 

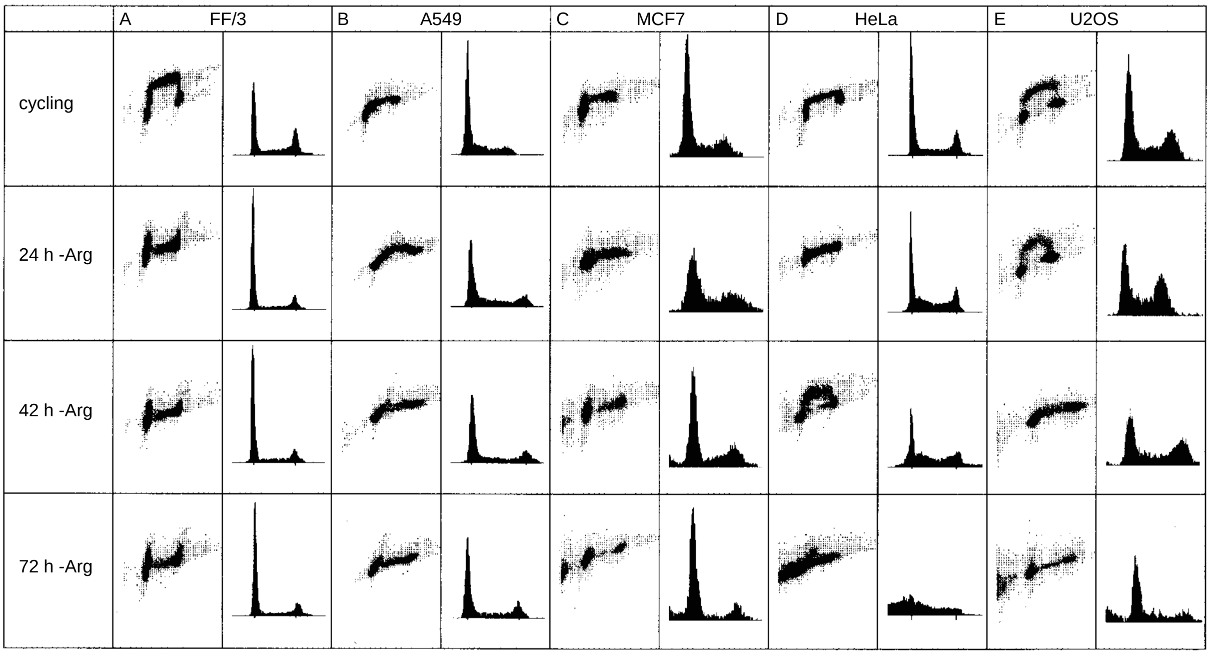

**Figure 7** Bivariate flow analysis of the effects of –Arg over the first 3 days of exposure relative to the normal cycling (+Arg) controls for five representative cell lines of those shown in Table 1. While normal cells (FF5/15) in S-phase manage to scavenge enough free Arg to move through S, G2 and M back into G1, no cells move from G1 into S-phase labelling (shown by the absence of the elevated part of the 'horseshoe' distribution on left, top of column **A**), A549 (**B**) remained almost unchanged in the frequency distribution profile, i.e. in a cell-cycle 'freeze', in which by 48 h they were not actively synthesizing DNA. MCF7 cells (**C**) did slowly move on through cycle, but by 72 h there was a noticeable increase in the debris to the left, as also in the case of HeLa (**D**). HeLa continued to synthesize DNA, which was to their severe detriment by 72 h. In U2OS (**E**), a similar pattern to HeLa cells was found

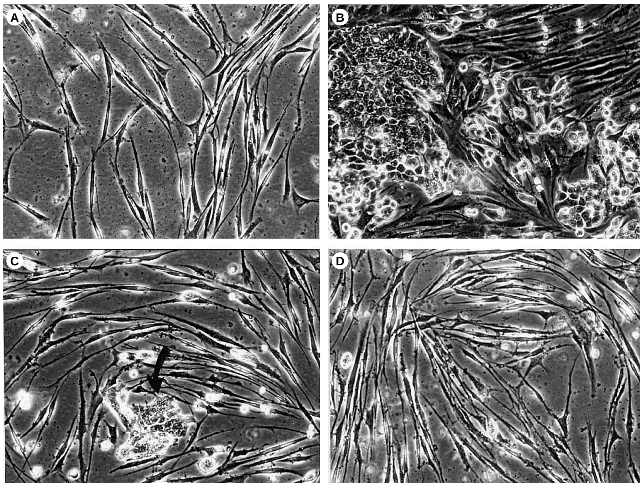

**Figure 8** Co-cultures of MGF2 and WiDr cells were exposed to medium with and without Arg. (**A**) The fibroblasts were set out at this density and seeded with WiDr cells to grow for 3 days, by which time the co-cultures looked like (**B**). At this time, half the cultures were switched to –Arg medium, and the other half continued in fresh +Arg medium. The latter co-cultures overgrew and could not be sustained more than 2–3 days more, with little change in from (**B**). In (**C**), the co-culture had been in –Arg medium for 3 days, at which time only a few small 'nests' (arrow) of tumour cells were left. Cultures which had reached state (**C**) were given +Arg medium and a further 2 days later (**D**) we see fibroblast regrowth and no evidence of any sustained WiDr repopulation. Magnification × 125

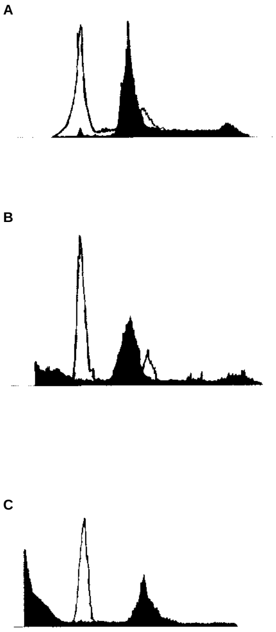

**Figure 9** Evidence of selective elimination of B16-F10 cells co-cultured with FF5/15 fibroblasts by flow cytometry. Twice as many melanoma cells as fibroblasts were seeded into cultures and examined after establishment as a 50–60% confluent monolayer within 2 days (**A**). After 36 h in –Arg, the B16-F10 pattern had radically altered (black), and showed signs of degeneration (particles towards the ordinate axis), whereas the fibroblasts had left S and accumulated mostly in G1, although a good G2 peak was also seen at this time. (**B**) By 72 h in –Arg, the fibroblasts were almost exclusively in G1, whereas some B16-F10 cells remaining intact occupied largely their G1 position, but the majority had degenerated. (**C**) By 5 days, all the B16-F10 had become small debris particles close to the ordinate.

*© 2000 Cancer Research Campaign*

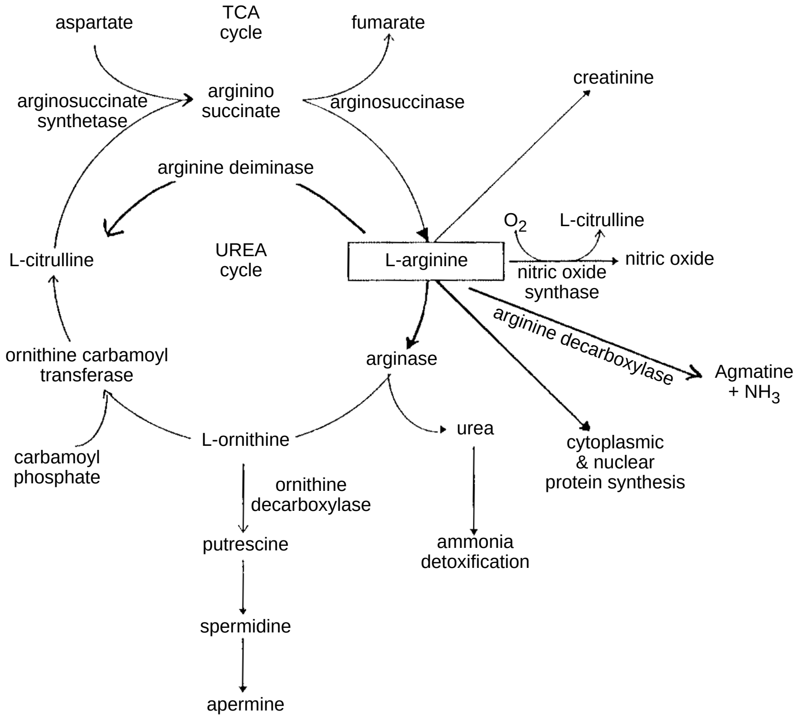

**Figure 10** Schema of some of the more typical involvements of arginine in cell metabolism. The frequent use of arginine as a source of precursor in pathways leading towards pyrimidine and purine synthesis have not been included seen in cell-cycle dynamics and distributions in this study. In the two 'resistant' malignant cell lines, A549 and MCF7 (see also Figure 3E and 3F for the latter), a cell-cycle 'freeze' was confirmed by their flow cytometric analysis at 24, 48 and 72 h of exposure (Figure 7B and 7C). The cells could be recovered after 10 days in –Arg, although by that time cell number had inevitably fallen slightly. After the rescued cells had grown to 60% confluence, they were exposed to a second episode of –Arg, to which they responded in the same manner as to the first episode, although a smaller percentage of cells survived for as long as 10 days following the second episode.

While the A549 and MCF7 cell lines showed arrest, it was by no means as clean as arrest of normal human fibroblasts which classically piled up in G. However, the fact that these tumour lines can stop all around the cycle contrasts strongly with virtually all the other malignant cell lines, which rapidly died. The paradigm here is HeLa (Figure 7D), to which similar traces were recorded for A431, B16 F10, WiDr and the many other established tumour cell lines. The death of these cells occurs as a consequence of their remaining in cycle with greatly protracted S- and G2-phases, unable to complete S-phase and dying in a premitotic catastrophe.

### Co-cultures

Selective death of malignant cell type was most readily apparent in co-cultures (Figure 8, Warrington, 1978). More than six different combinations of malignant and normal cells have been admixed, and survival of both malignant and normal cells only observed when the former were not susceptible to cell death under –Arg conditions (e.g. A549 co-cultured with normal diploid fibroblasts). We have taken seeding ratios as high as 8 times the malignant cell line to normal cells, and found that the former were almost completely decimated within 3–4 days, with virtually no loss of fibroblasts. A typical result from an admixture of roughly equal (starting) numbers of human fibroblasts (FF5/15) with B16–F10 melanoma cells has also been followed using flow cytometry, since the frequency distribution patterns are quite distinct and clearly show the selective death of the tumour cell line (Figure 9).

### DISCUSSION

### Responsiveness to Arg deprivation compared with other amino acid deficiencies

Analysis of the effects of deprivation of each of the eleven essential amino acids in turn on the number of lines used in this study would be prohibitively burdensome, but we have in fact checked many besides Arg. The data so far continues to be fully consistent with our previous findings (Wheatley et al, 2000), lending further support to the idea that most tumour cell lines die considerably more quickly during Arg deprivation than in the absence of any other single (or group of) essential amino acids. For this reason, we focused exclusively on Arg deprivation in the present study. Furthermore, Arg begins to become limiting at ~$10^{-4}$ M, whereas other amino acids can sustain growth at $10^{-5}$–$10^{-6}$ M (Wheatley et al, 2000).

### Loss of viability associated with checkpoint derangement in tumour lines

We have shown (Wheatley et al, 1993; 2000) that the loss of viability in most of the malignant phenotypes reflects derangement

© 2000 Cancer Research Campaign of control primarily at the key G1 checkpoint, which normally prevents cells from reinitiating DNA synthesis under adverse circumstances. In the majority of transformed cell lines, i.e. other than our two notable exceptions, cells which nevertheless remained intact after 1 week of deprivation were invariably multinucleated large cells which were irrecoverable in +Arg medium. Thus, not only is the *overwhelming majority of tumour lines* susceptible to Arg deprivation, but *cell death occurs without any further intervention*. Our two exceptional lines will, however, prove invaluable in pinpointing the molecular aberrations of the cell-cycle engine responsible for the demise of the susceptible types (see below).

Our findings with 3T3 cells fully support those of Tanaka et al (1988), who showed that NIH 3T3 cells die in Arg deprivation, as do supposedly related 3T3 'sublines' (Table 1). Since 3T3 cells are so widely used in serum step-down cell-cycle studies, their response to amino acid deprivation indicates a lack of R-point stringency, although the ability to induce synchrony following serum deprivation has always suggested otherwise. Thus the requirement for serum factors may be more crucial for the movement of transformed cells from G1- into S-phase than the presence of essential amino acids, which is in keeping with the ability of other established cell lines to be parasynchronized by serum deprivation (Rubin and Xu, 1991; Yin and Wheatley, 1994), but interestingly not by amino acid withdrawal. In well-regulated cells, serum and amino acid deprivation are supposed to map temporally to the same G1 point (Yen and Pardee, 1978; Lamb and Wheatley, 2000), but these findings suggest otherwise.

### Cycling differences shown by flow cytometry

'Obedient' cells placed in arginine-deficient medium are either arrested within about 5–6 h from further entry into S-phase (Lamb and Wheatley, 2000), or if they have already passed this point, they complete the ongoing cycle and enter quiescence (G0), usually within ~24–30 h. The prolonged survival (>4 weeks) of normal cells (human diploid fibroblasts) is remarkable, although an initial cell drop-out of ~10% of the total population is seen within the first 48 h of deprivation, followed by a much slower, almost imperceptible, decline in total cell number thereafter. In contrast, susceptible cells continue to re-enter S-phase, and most of those already started never complete it. This agrees with the findings of Weissfeld and Rouse (1977*a–c*) on KB and CHO cells. Bivariate flow cytometric analyses (Figure 7) are consistent with these observations. No cells enter mitosis after even a few hours of being deprived (Lamb and Wheatley, 2000; Wheatley et al 2000). That progression to cell death out of S-phase or G2-phase occurs by way of a premitotic catastrophe, nevertheless, is conjecture and needs further elucidation.

Loss of constraint at the G1 checkpoint results in continued cdk4-cyclin D-mediated phosphorylation of retinoblastoma protein (pRb). In a previous paper, we have described in detail the dependence of R-point control on the phosphorylation of pRb by active cdk4-cyclin D complexes in normal fibroblasts (Lamb and Wheatley, 2000). G1 arrest in response to a number of growth inhibitory conditions has been shown to be due to repression of cdk4 (Ando and Griffin, 1995). It is possible that p53 may be responsible for cdk4 downregulation in the absence of arginine, since a p53-dependent pathway has been identified in response to TGF-β-mediated G1 arrest (Ewen et al, 1993; 1995) and p53 is mutated in most tumour cells. We are currently assessing p53 functionality and determining the levels of cdk4 and p21 as a consequence of arginine deprivation, using suitable cell lines including many of those discussed herein and fresh primary tumour cell cultures (in preparation; *see* Lamb, 1998; Scott, 1999). The possibility must also be kept in mind that more than a single aberration in cell-cycle control will probably be present in highly malignant phenotypes. For example, it is well known that most tumours have mutant p53 (see below).

### Exceptions to the 'rule'

A549 and MCF7 did not show the same clean, debris-free appearance of normal cells in –Arg, the proportion of cells dropping out before the real survivors being considerably greater . Nevertheless, their ability to survive does not mean that they are devoid of defective control at cycle checkpoints other than the R-point, just as the other (susceptible) tumour cells may have multiple derangements. The nature of their 'cell-cycle freeze' needs further investigation, but it is possible that they avoid mitotic catastrophe by retaining late-cycle checkpoint stringency, lost in most other tumour cell types examined. The possibility that the resistant cells have normal (or at least functional) p53 status has been explored (Scott, 1999), and indeed preliminary data shows that both A549 and MCF7 have functional p53, unlike the situation in sensitive cell lines (Scott et al, 1998). The notion, therefore, is that they could be avoiding the hazard that their mitotic impetus is driving them into the cell death pathway because they do slowly filter through to G1 of the next cycle.

### Implications of selective tumour cell destruction for cancer therapy

Arg is the first amino acid to be exhausted by normal cell metabolism in culture (Hanss and Moore, 1964). The pathways for which it is the first or an early substrate are diverse, as shown by a diagram of its major metabolic involvements (Figure 10). To date we have explored all the essential amino acids using HeLa cells as the tumour cell assay (Wheatley et al, 2000), although it remains a possibility that different cell lines are especially sensitivity to the lack of certain other amino acids. Methionine can be taken as a case in point, as shown by Guo et al (1993). However, we are in general confident that arginine deprivation works better in almost all tumour lines, and the future strategy for improved cancer treatment will be based specifically on its withdrawal. While this can easily be achieved in vitro, the same is not true of the in vivo situation. Nevertheless, if it can be achieved, a clear practical advantage can be gained for a more rational and selective therapeutic approach to cancer treatment. An added bonus is that, not only does the poorer regulation of the R-point in malignant cells help to selectively target cancer cells, but that a remarkably high death rate follows naturally within a few days of deprivation *without the need for any further intervention*.

Some oncologists have recommended Arg *supplementation* to enhance cell-kill rate during chemotherapy (Brittenden et al, 1994), supposedly through boosting host immunosurveillance mechanisms (Hester and Fee, 1995). The case is not proven, and therefore more consideration ought perhaps be given to arginine *deprivation*, which has had a following in the past (Wheatley,

*© 2000 Cancer Research Campaign*

1998). While sensitive tumour cells quickly succumb, we have considered combination treatment for resistant cell types. This we hope to achieve through the use of very low levels of cycle-dependent DNA-targeting drugs since the resistant tumour cells, unlike normal cells, remain in cycle with a preponderance of cells in S-phase. Our initial findings confirm previous work that an adequately low-dose level must be carefully chosen, because there comes a point at which the concentration of a DNA-damaging cell-cycle-dependent drug will itself slow down or prevent the re-initiation of S-phase in cells which would otherwise have moved on past the G1 checkpoint. Counterproductively, this introduces an element of protection rather than an augmentation or enhancement of the cell-killing effect of –Arg alone (Lamb and Wheatley, 1996). It will therefore be of interest to see whether Arg-deprived MCF7 and A549 cells succumb to combined low-dose therapy (Stirrat et al, submitted; and work in progress).

Some investigators have advocated the combined use of analogue amino acids (Ryan and Elliott, 1968; Warrington, 1992; Rabinovitz, 1996). These offer no further benefit because they affect normal cells as much as malignant cells by their incorporation into proteins (Wheatley et al, 2000). Treatment with canavanine and *p*-fluorophenylalanine in –Arg and –Phe conditions respectively actually obfuscate the selectivity gained with –Arg treatment alone.

In conclusion, lack of Arg alone has such a marked effect on tumour cells that augmentation with other modalities of treatment become unnecessary. Most tumour cells die quickly, whereas normal cells enter quiescence and survive for long periods of time. Some tumour lines (~10%; e.g. A549 and MCF7 cells) appear to downregulate activity in response to –Arg, but may enter a cell-cycle freeze. The sensitivity of primary tumours can therefore be screened in vitro by testing with the various techniques employed herein, to see whether they are sensitive or resistant to Arg deprivation. If this procedure can be translated to the in vivo situation, control of Arg levels in animal and human tumour patients should bring about the demise of cancer even in disseminated (metastatic) disease.

### ACKNOWLEDGEMENTS

We thank Mrs Sandra Latter for her help with culturing stock cells, and testing each one for contamination with mycoplasma before experimentation. The cells lines have been verified as of the correct characterization before use, and used at the earliest possible passage number from stock, in accord with the recommended practice in the *UKCCCR Guidelines*. This work was supported in part by Swiss National Science Foundation, The AO Institute (Davos) and Cancer Treatments International (Zürich). We particularly wish to thank our Swiss colleagues, Dr Slobodan Tepic and Professor Pierre Montavon (Tierspital, Zürich) for their encouragement, help, and invaluable advice; also Professor Arthur Pardee (Dana Farber Cancer Institute, Boston) for his continued interest in this work.

### REFERENCES

Ando K and Griffin JD (1995) Cdk4 integrates growth stimulatory and inhibitory signals during G1 phase of hematopoietic cells. *Oncogene* **10**: 751–755

Bach SJ and Swaine D (1965) The effect of arginase on the retardation of tumour growth. *Br J Cancer* **19**: 379–386

Brittenden J, Heys SD and Eremin O (1994) L-arginine and malignant disease: a potential therapeutic role? *Eur J Surg Oncol* **20**: 189–192

Daniel JC and Krishnan RS (1968) Amino acid requirements for growth of the rabbit blastocyst *in vitro*. *J Cell Physiol* **70**: 1550–1560

Dolbeare F, Gratzner H, Pallavicini MG and Gray JW (1983) Flow cytometric measurement of total DNA content and incorporated bromodeoxyuridine. *Proc Natl Acad Sci USA* **80**: 5573–5577

Eagle H (1955) Nutritional needs of mammalian cells in tissue culture. *Science* **122**: 501–504

Eagle H (1959) Amino acid metabolism in mammalian cell cultures. *Science* **130**: 432–437

Ewen MT, Sluss HK, Sherr CJ, Matsushine H, Kato J and Livingstone DM (1993) Functional interactions of the retinoblastoma protein with mammalian D-type cyclins. *Cell* **73**: 487–497

Ewen ME, Oliver CJ, Sluss HK, Miller SJ and Peeper DS (1995) P53-dependent repression of CDK4 translation in TGF-β-induced G1 cell cycle arrest. *Genes Dev* **9**: 204–217

Guo H, Lishko VK, Herrera H, Groce A, Kubota T and Hoffman RM (1993) Therapeutic tumor-specific cell cycle block induced by methionine starvation *in vivo*. *Cancer Res* **53**: 5676–5679

Hanss J and Moore GE (1964) Studies of culture media for the growth of human tumor cells. *Exp Cell Res*, **34**: 242–256

Hester JE and Fee WE (1995) Effect of arginine on growth of squamous cell carcinoma in the CH3/KM mouse. *Arch Otolaryngol Head Neck Surg* **121**: 193–196

Lamb J (1998) *Molecular aspects of amino acid sensitive cell cycle control*. PhD Thesis. University of Aberdeen: Aberdeen

Lamb J and Wheatley DN (1996) Cell killing by the novel imidazoacridinone antineoplastic agent, C-1311, is inhibited at high concentrations coincident with dose-differentiated cell cycle perturbation. *Brt J Cancer* **74**: 1359–1368

Lamb J and Wheatley DN (2000) Single amino acid (arginine) deprivation induces G1 arrest associated with inhibition of cdk4 expression. *Exp Cell Res* (In press)

Mosmann T (1983) Rapid colorimetric assay for cellular growth and survival: application to proliferation and cytotoxicity assays. *J Immunol Methods* **65**: 55–63

Neff NT, Ross PA, Bartholomew JC and Bissell MJ (1977) Leucine in cultured cells. *Exp Cell Res* **106**: 175–183

Ormerod MG, Orr RM and Peacock JH (1994) The role of apoptosis in cell killing by cisplatin; a flow cytometric study. *Br J Cancer* **69**: 93–100

Pardee AB (1974) A restriction point for control of normal animal cell proliferation. *Proc Natl Acad Sci USA* **71**: 1286–1290

Pardee AB (1989) G1 events and the regulation of cell proliferation. *Science* **246**: 603–608

Paul D and Walter S (1974) Growth control in primary fetal rat liver cells in culture. *J Cell Physiol* **85**: 113–124

Rabinovitz M (1992) The pleiotypic response to amino acid deprivation is the result of interactions between components of the glycolysis and protein synthesis pathways. *FEBS Lett* **302**: 113–116

Rabinovitz M (1996) Unchanged tRNA-phosphofructokinase interaction in amino acid deficiency. *Amino Acids* **10**: 99–108

Rubin H and Xu K (1991) Epigenetic features of spontaneous transformation in the NIH 3T3 line of mouse cells. In *Boundaries between promotion and progression during carcinogenesis*, O. Sudilovsky et al (eds), pp 301–313 Plenum Press: New York

Ryan WL and Elliott JA (1968) Fluorophenylalanine inhibition of tumors in mice on a phenylalanine-deficient diet. *Arch Biochem Biophys* **125**: 797–801

Scott LA (1999) *Arginine deprivation and tumour cell death*: in vitro *and* in vivo *studies*. Ph.D.thesis. University of Aberdeen: Aberdeen

Scott LA, Lamb J and Wheatley DN (1998) Defects of cell cycle control in malignant cells causes rapid death in arginine deprivation. *Mol Cell Biol Suppl* 10: Abst. 1572

Storr JM and Burton AF (1974) The effects of arginine deficiency on lymphoma cells. *Br J Cancer* **30**: 50–59

Tanaka H, Zaitsu H, Onodera K and Kimura G (1988) Influence of the deprivation of a single amino acid on cellular proliferation and survival in rat 3Y1 fibroblasts and their derivatives transformed by a wide variety of agents. *J Cell Physiol* **136**: 421–430

Tobey RA and Ley KD (1970) Regulation of the initiation of DNA synthesis on Chinese hamster cells. 1. Production of stable, reversible G1-arrested populations in suspension culture. *J Cell Biol* **46**: 151–157

Warrington RC (1978) Selective killing of oncogenic human cells cocultivated with normal human fibroblasts. *J Natl Cancer Inst* **61**: 69–73

Warrington RC (1992) L-histidinol in experimental cancer chemotherapy – improving the selectivity and efficacy of anticancer drugs, eliminating metastatic disease and reversing multidrug-resistant phenotype. *Biochem Cell Biol* **70**: 365–375

Weissfeld AS and Rouse H (1977*a*) Continued initiation of DNA synthesis in arginine-deprived Chinese hamster ovary cells. *J Cell Biol* **73**: 200–205

Weissfeld AS and Rouse H (1977*b*) Arginine deprivation in KB cells. I. Effect on cell cycle progress. *J Cell Biol* **75**: 881–888

Weissfeld AS and Rouse H (1977*c*) Arginine deprivation in KB cells. II. Characterization of the DNA synthesized during starvation. *J Cell Biol* **75**: 889–898

Wheatley DN (1998) Dietary restriction, amino acid availability and cancer. *The Cancer Journal* **11**: 183–189

Wheatley DN, Miseta A, Love EM, Strickland D and Harris I (1993) Effect of the immediate precursors of phenylalanine and tyrosine on growth and protein synthesis in phenylalanine- and tyrosine-deprived HeLa cells. *Biochim Biophys Acta* **1164**: 209–214

Wheatley DN, Scott L, Lamb J and Smith S (2000) Single amino acid (arginine) restriction: growth and death of cultured HeLa and human diploid fibroblasts. *Cell Physiol Biochem* (In press)

Wu G and Morris SM Jr (1998) Arginine metabolism: nitric oxide and beyond. *Biochem J* **336**: 1–17

Yeatman TJ, Risley GL and Brunson ME (1991) Depletion of dietary arginine inhibits growth of metastatic tumor. *Arch Surg* **126**: 1376–1382

Yen A and Pardee AB (1978) Arrested states produced by isoleucine deprivation and their relationship to the low serum produced arrested state in Swiss 3T3 cells. *Exp Cell Res* **114**: 389–395

Yin Z and Wheatley DN (1994) Sensitivity of 3T3 cells to low serum concentration and the associated problems with serum withdrawal. *Cell Biol Int* **18**: 39–47

*© 2000 Cancer Research Campaign*
